# Luminescent AIE Dots for Anticancer Photodynamic Therapy

**DOI:** 10.3389/fchem.2021.672917

**Published:** 2021-05-25

**Authors:** Zhenyan He, Sidan Tian, Yuting Gao, Fanling Meng, Liang Luo

**Affiliations:** ^1^National Engineering Research Center for Nanomedicine, College of Life Science and Technology, Huazhong University of Science and Technology, Wuhan, China; ^2^Hubei Key Laboratory of Bioinorganic Chemistry and Materia Medica, School of Chemistry and Chemical Engineering, Huazhong University of Science and Technology, Wuhan, China

**Keywords:** photodynamic therapy, photosensitizers, aggregation-induced emission, AIE dots, theranostics

## Abstract

Photodynamic therapy (PDT) is an emerging effective strategy for cancer treatment. Compared with conventional cancer therapies, such as surgery, chemotherapy, and radiotherapy, PDT has shown great promise as a next-generation cancer therapeutic strategy owing to its many advantages such as non-invasiveness, negligible observed drug resistance, localized treatment, and fewer side effects. One of the key elements in photodynamic therapy is the photosensitizer (PS) which converts photons into active cytotoxic species, namely, reactive oxygen species (ROS). An ideal PS for photodynamic therapy requires the efficient generation of ROS, high stability against photo bleaching, and robust performance in different environments and concentrations. PSs with aggregation-induced emission (AIE) characteristics have drawn significant attention, in that they can overcome the aggregation- caused quenching effect that is commonly seen in the case of fluorescence dyes and provide excellent performance at high concentrations or in their condensed state. Moreover, organic nanomaterials with AIE characteristics, or AIE dots, have played an increasingly significant role in assisting PDT based on its excellent ROS generation efficiency and simultaneous imaging feature. This review summarizes the recent advances on the molecular design of AIE PSs and AIE dots-based probes, as well as their emerging applications for enhanced anticancer PDT theranostics.

## Introduction

Cancer is one of the leading causes of death worldwide. Owing to its increasing incidence and constantly high mortality, cancer is becoming a major health problem for the public (Siegel et al., 2016, 2017, 2018; Small et al., 2017). In the fight against cancer, researchers have been focusing on different strategies to control tumor growth and improve the quality of life of patients. However, after years of struggle, surgery still remains the first choice for cancer treatment (Best et al., 2016; Miller et al., 2016; Benitez Majano et al., 2019). Non-invasive therapies, such as radiotherapy (RT) and chemotherapy, are severely limited by serious systemic side effects on normal tissues, and their effects are still far from satisfactory (Dong et al., 2007a; Johnstone et al., 2013; Naik et al., 2014). Therefore, novel non-invasive therapies that can efficiently kill cancer cells with minimized side effects are urgently needed (Kim et al., 2009; Veiseh et al., 2010; Lucky et al., 2015).

Among numerous conceivable approaches, photodynamic therapy (PDT) is a non-invasive treatment that employs light irradiation to kill cancer cells and holds unique advantages over other methods (Schuitmaker et al., 1996; Oleinick et al., 2002). Similar to the more conventional RT, PDT offers good spatiotemporal control by tunable irradiation (Henderson and Dougherty, 1992). On the other hand, compared with harmful radiation, the use of non-cytotoxic visible or near infrared light raises an opportunity to reduce the damage on normal tissues (Oleinick et al., 2002; Brown et al., 2004). Photosensitizer (PS) is the key element that converts photons to reactive oxygen species (ROS). A PS can generate different types of ROS, such as singlet oxygen (^1^O_2_), H_2_O_2_, ${\text{O}}_{2^{-•}}$, and ^·^OH, upon light irradiation (Dolmans et al., 2003). An ideal PS for PDT should be biocompatible, causing no harm to cells in the dark and should efficiently generate ROS to kill cancer cells upon irradiation. In addition, a powerful PS should be stable and easy to accumulate in tumor tissues. Finally, a good PS enables long wavelength excitation for better tissue penetration (Escobedo et al., 2010).

To date, researchers have already made tremendous efforts to develop PSs that meet the above criteria (Henderson and Dougherty, 1992); however, due to their hydrophobic and rigid planar structures, the aggregation-caused quenching (ACQ) effect remains a great obstacle to their usage (Yuan et al., 2014; Gao et al., 2017). Traditional PSs show significant fluorescence quenching effect and reduction of ROS generation in their aggregated state, which will compromise the quality of fluorescence imaging and the effect of PDT (Park et al., 2011; Sekkat et al., 2011; Rajaputra et al., 2013; Zhang et al., 2014; Zheng X. et al., 2017). Therefore, they can only be used in good solvents and at low concentrations, which greatly limits the utilization of many promising PSs for PDT applications. A number of studies have been conducted to suppress the ACQ phenomenon by physical, chemical, and engineering approaches (Gaylord et al., 2001; Chiang et al., 2008). However, PSs with an intrinsic strong fluorescence and high quantum yield of reactive oxygen species generation in their aggregated states are still highly needed.

**Figure 1:**
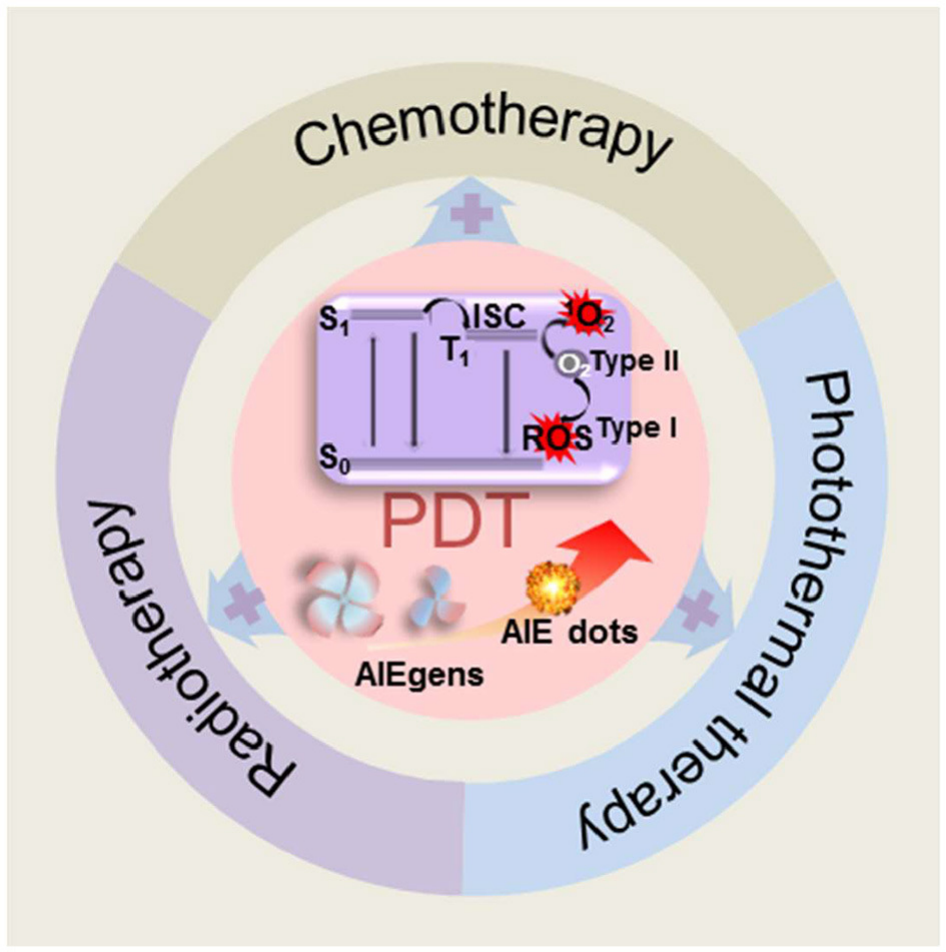
Schematic illustration of dots or nanomaterials based on AIEgens for photodynamic therapy applications.

Luo et al. (2001) reported an interesting aggregation-induced emission (AIE) molecule, whose emission was weak in solutions but dramatically enhanced in aggregates. Later, many AIE luminogens (AIEgens) have been synthesized and widely applied for different purposes (Dong et al., 2007b; Tong et al., 2007; Chen et al., 2010; Kwok et al., 2015; Sun et al., 2018). AIEgen is a powerful material to solve issues caused by the ACQ effect and allows for a fluorophore to be used in its aggregated state or at high concentrations, which is very beneficial for PDT applications due to their advantages of high quantum yield of ROS generation, optimal biosafety, structural diversity, tunable properties, and robust reproducibility (Hsieh et al., 2013; Ji C. et al., 2018). AIEgen-based PSs are therefore regarded as one of the most promising candidates for future PDT applications; however, most AIE PSs are highly hydrophobic, which severely limits their biological applications by binding with biological molecules. Developing drug delivery systems to improve the PDT efficiency of AIE PSs is considered a viable approach. Nanomaterials, such as inorganic nanomaterials, liposomes and nanocrystals, have excellent advantages in biological applications. They can target tumor sites through the enhanced permeability and retention (EPR) effect *via* intravenous injection and diagnose and treat tumor sites precisely. Among them, nanomaterials modified by polypeptides that target tumor biomarkers can encapsulate drugs (such as AIE PSs) and actively transport the AIE PSs to the tumor site. Inorganic nanomaterials, such as quantum dots and carbon nanotubes (CNTs), have tunable emission wavelengths with high brightness (Juang et al., 2020); however, they are potentially toxic. In contrast, organic nanomaterials are more suitable for biomedical applications. As we have known, nanomaterials composed of aggregates of AIEgens (AIE dots) have also been developed for tumor imaging, disease diagnosis, drug delivery monitoring, image-guided cancer treatment, among other processes. AIE dots are particularly attractive because they have the characteristics, both of AIEgen and nanomaterials, that are favorable for drug delivery, imaging, and PDT (Guo et al., 2020; Huang et al., 2020; Song et al., 2020). Moreover, AIE nanoparticles (AIE NPs) perform well in prolonging the blood circulation time and improving therapeutic efficacy; attributed to their ordered architectures, tailored morphologies, controllable functions, and EPR effect. In this review, we will summarize current AIE dots, categorized by structure modalities, that have been utilized to enhance the performance in anticancer PDT (Figure 1). Combined PDT therapies involving AIE dots and other therapeutics will also be discussed.

## AIE Dots Assisting Anticancer PDT

Photosensitizer is the key element in PDT (Zhou et al., 2016). In a typical PDT process, after absorbing the photons, PSs are excited to the excited singlet state (S_1_) from the ground state (S_0_). When the excited electrons return to the ground state *via* releasing either non-radioactive energy or radiative energy as fluorescence, part of them in S_1_ is transferred to the triplet state (T_1_) by intersystem crossing (ISC). The energy is transferred to triplet oxygen to generate highly active ^1^O_2_ (type II reaction), which can efficiently damage tumor cells. Due to the hypoxic nature of solid tumors, consumption of O_2_ during the PDT process aggravates the O_2_ shortage and inhibits PDT efficiency. To address this issue of hypoxia, various approaches have been proposed to enhance PDT efficacy, such as external O_2_ supply and type I reaction (Gao et al., 2020). In type I reaction, the PS in the T_1_ state can destroy tumor cells by transferring electrons to the surrounding substances to form cytotoxic species such as free radicals and ROS, without O_2_ dependency (Zhou et al., 2016). The reported study proved that tuning D–A structures to balance charge transfer and local excitation states is an effective strategy to improve the yield of the triplet state. Moreover, an ideal moiety that can stabilize an external electron to form radical anion intermediates is also beneficial. For example, phosphindole oxide (PIO), which can form radical anion intermediates, has been reported as an electron-accepting building block to construct donor–acceptor (D–A) to trigger dual cell death modes of apoptosis and autophagy *via* the type I mechanism (Zhuang et al., 2020). As we have mentioned above, AIE PSs are particularly efficient since they can be used at high concentrations and exhibit an enhanced stability. Numerous studies have been carried out to generate AIE PSs with different structures and functions (Yuan et al., 2014; Wang et al., 2018; Deng et al., 2020; Wu et al., 2021). Besides the inherent ability of AIE PSs to generate ROS, they can also be covalently linked to conventional PSs, so that the newly formed PSs can overcome the major drawbacks of the ACQ effect, and can be used at high concentrations or in different aggregated states. More interestingly, AIE PSs with “switch-on” properties offer an easy access to the realization of tunable and stimuli-responsive “smart” PDT in cancer treatment. Among AIE PSs employed in anticancer PDT, tetraphenylethylene and triarylamine are the two major molecular core structures. In this study, we summarize recent related reports on structural aspects of AIE PSs.

## Tetraphenylethylene and Derivatives

**Figure 2:**
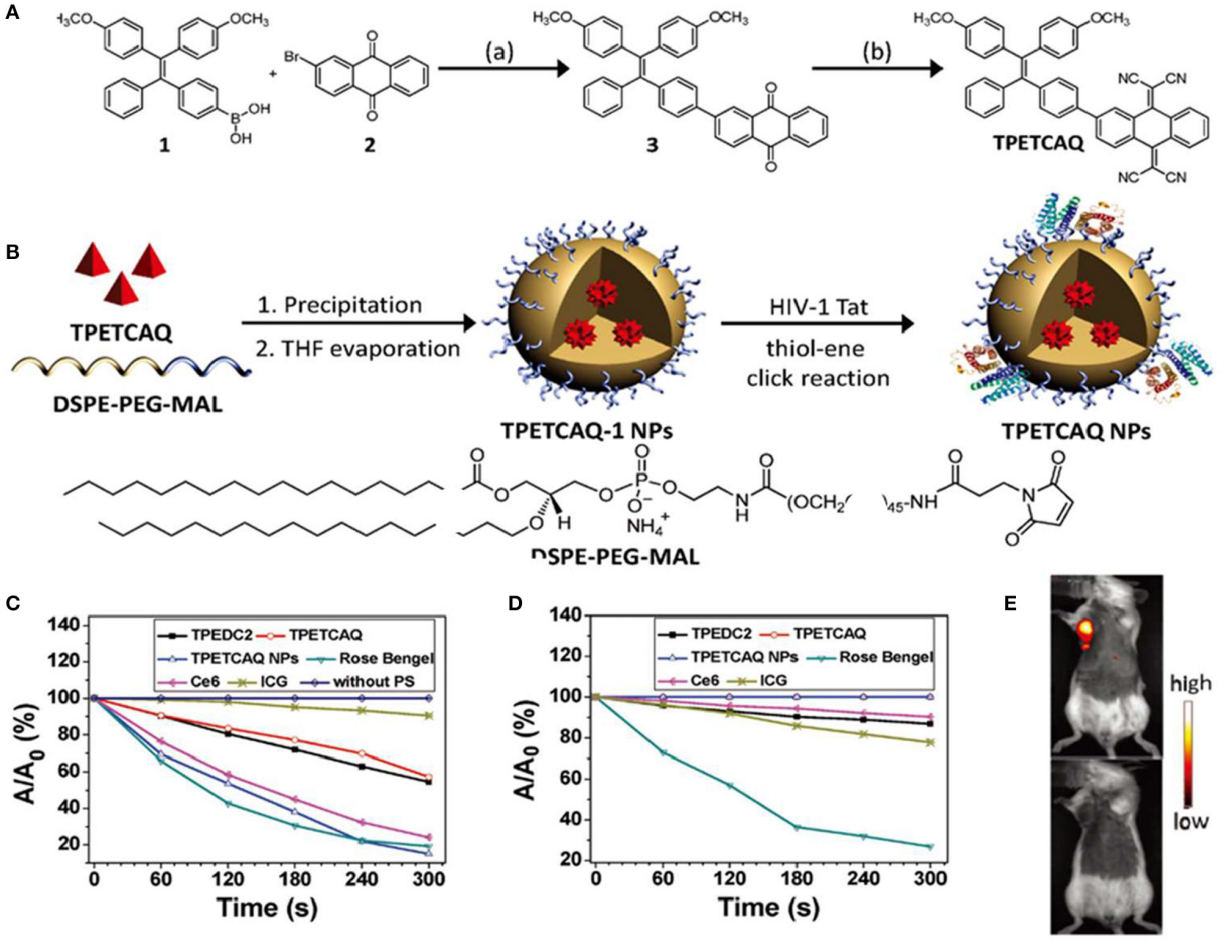
**(A)** Synthetic route and chemical structure of TPETCAQ. **(B)** Synthesis of TPETCAQ-1 and TPETCAQ nanoparticles (NPs). **(C)** Decomposition rates of ABDA in the presence of different PSs under light irradiation. **(D)** Decomposition rates of different PSs under light irradiation. **(E)** Fluorescence imaging of a 4T1-Luc tumor bearing mice after intratumoral administration of either TPETACQ NPs (30 μl of 1 mg/ml for TPETCAQ, top) or saline (bottom) at 1 h after injection. Reproduced with permission from Xu et al. (2015). Copyright 2015 Wiley Online Library.

Tetraphenylethylene (TPE) and its derivatives have been studied as AIE core structures, attributing to their excellent AIE properties (Wang et al., 2010; Zhao et al., 2012). Moreover, it is easy to synthesize and functionalize TPE with different functional groups (Dong et al., 2007b). Recent studies have demonstrated a number of functionalized TPE-based PSs with efficient ROS generation. For example, a TPE-based PS, named TPETCAQ, was synthesized as shown in Figure 2A; however, due to intramolecular charge transfer, the fluorescence enhancement and ROS quantum yield of TPETCAQ in polar aqueous solutions are not significant (Wu et al., 2017). To overcome this problem, TPETCAQ was loaded in polymeric nanoparticles (Figure 2B). As a result, the fluorescence intensity and ROS quantum yield of TPETCAQ were significantly enhanced, as shown in Figures 2C,D. These nanomaterials were further functionalized with a cell penetrating peptide HIV-1 Tat to aid their uptake into the cells. TPETCAQ is emitted at a wavelength of 820 nm, which allows for deep penetration in tissues. As shown in Figure 2E, after intratumor injection of TPETCAQ NPs (30 μl, 1 mg/ml), NIR fluorescence was observed at the tumor site, indicating good imaging performance of TPETCAQ NPs *in vivo*. As the incubation time increased, tumor cells were killed by the resulting ROS, and the fluorescence at the tumor site almost disappeared. The correlation between the fluorescence intensity and the tumor volume indicated an excellent NIR imaging-guided tumor elimination (Xu et al., 2015).

In another study with TPE derivatives, the authors described TRGF-NQ-cRGD as a non-phototoxic molecule, as its fluorescence quenching moiety allowed no fluorescence in the pristine state (Gu et al., 2017). When the linker was cleaved by biomolecules containing thiols, the fluorescence-quenching moiety was separated from the TPETF bearing the RGD group, leading to the burst of the fluorescence intensity. With increased incubation times with glutathione (GSH), the fluorescence intensity gradually increased. Since the GSH concentration is usually high in cancer cells, this redox-responsive AIE molecule exhibits a selective “turn-on” in cancer cells. Similar to other AIE PSs, in the absence of GSH, TRGF-NQ-cRGD could not efficiently generate ROS and could not reduce the absorption of 9,10-anthracenediyl-bis(methylene)dimalonic acid (ABDA). With the addition of GSH, the released TPETF exhibited strong fluorescence and the ROS production was significantly enhanced. After its release, TPETF-NQ-cRGD could target cancer cells with overexpressed α_v_β_3_ integrin, such as U87-MG or MDA-MB-231 cells.

**Figure 3:**
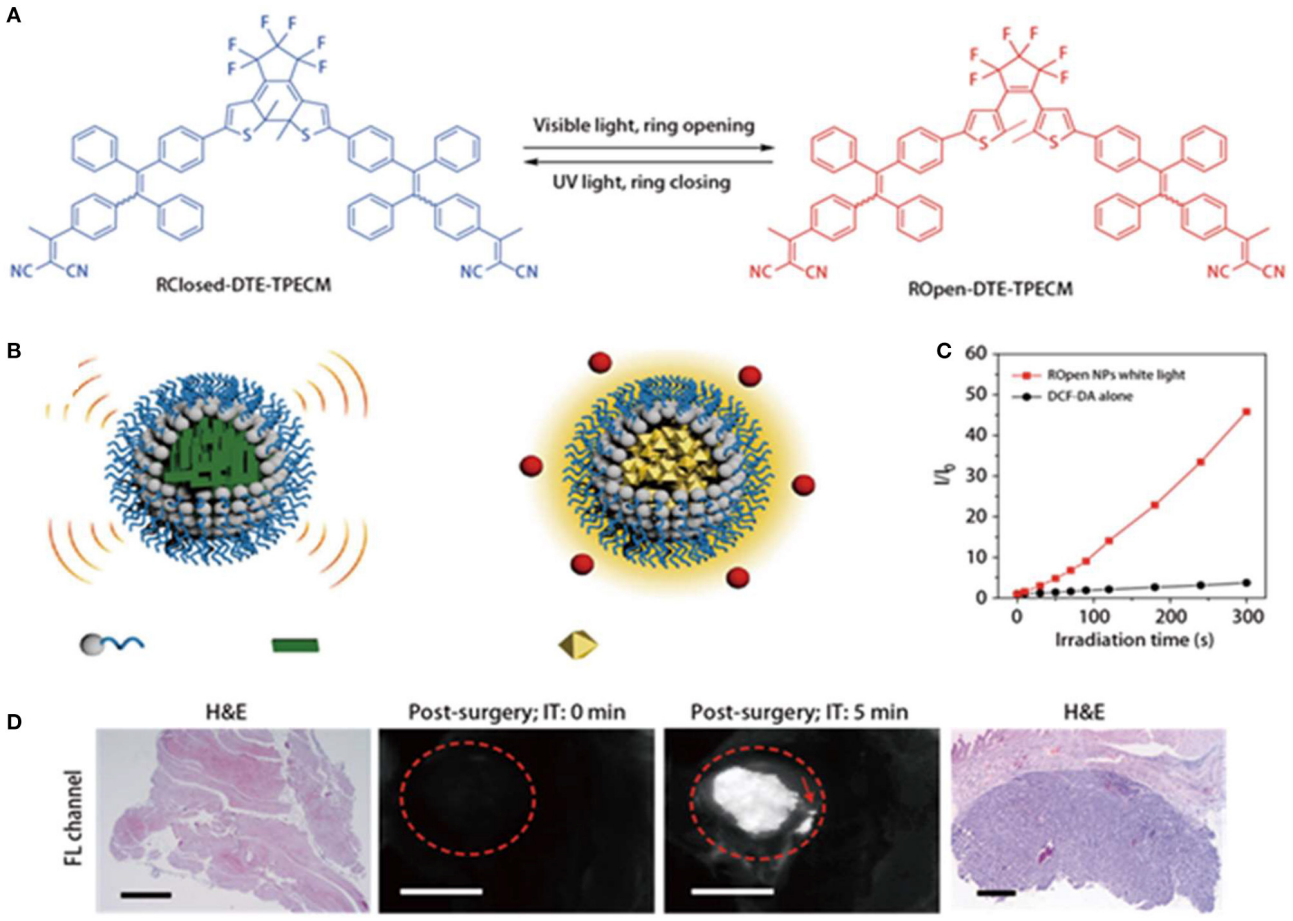
**(A)** Photo-controlled reversibility and optimized geometric structures of DTE-TPECM molecules. **(B)** Schematic representation of RClosed and ROpen NPs. **(C)** Plot of I/I_0_ vs. white light (0.25 W/cm^2^) irradiation time of ROpen NPs (10 μM based on ROpen-DTE-TPECM) in an aqueous solution. I_0_ and I are the PL intensities of dichlorodihydrofluorescein (DCF) at 525 nm before and after light irradiation at designated time intervals. **(D)** Representative fluorescence images of RClosed-YSA NP-treated mice with residual post-surgery tumors. The operative incision site was irradiated with 610 nm red light (0.3 W/cm^2^) for 5 min. The red dashed circles indicate the tumor/operative incision site. The red arrow shows the residual tumors with a diameter below 1 mm. The scale bars are 3 mm long. HandE-stained tissues at the operative incision site confirm the existence of residual tumors. Reproduced with permission from Qi et al. (2018). Copyright 2018 Nature Publishing Group.

Photoacoustic imaging and fluorescence imaging have their own advantages in terms of sensitivity and penetration depth (Liu et al., 2019). However, it is very challenging to develop materials that can simultaneously exert both effects. Qi et al. (2018) constructed smart function-transformable NPs based on the photo-controllable molecule (DTE-TPECM) for cancer diagnosis and treatment during surgery, as shown in Figure 3A. DTE-TPECM consists of a DTE core and two surrounding TPECM units and can occur as two isomers, namely, a ring-closed form and a ring-opened form. These two isomers are reversibly switchable upon external UV/visible irradiation. The fluorescence intensity of ROpen-DTE-TPECM increased in THF/water mixtures containing different water fractions due to the AIE effect of ROpen-DTE-TPECM. On the other hand, RClosed-DTE-TPECM possessed a more planar geometric structure, making it thermally inactive (Figure 3B). RClosed nanoparticles hardly generated any ROS upon excitation at either 365 or 610 nm. ROpen-DTE-TPECM significantly suppressed the non-radiative decay pathways; thus, the fluorescence emission and ROS generation were both enhanced (Figure 3C). To achieve a good biocompatibility and targeting, the molecule was loaded in a nanocarrier and coupled to a targeting peptide selectively bound to the EphA2 receptor, which was overexpressed on the cancer cell membranes. Therefore, the converted ROpen-YSA NPs have good ROS generation upon white light irradiation, while photoacoustic imaging could be achieved at 610-nm light irradiation without any cytotoxicity *in vitro*. ROpen-YSA NPs not only produced ROS for PDT but also provided a good diagnostic signal by means of RClosed-YSA NPs in surgical treatment (Figure 3D).

## Triarylamine and Derivatives

Triarylamine (TAA) and its derivatives are also commonly used as AIE PSs in PDT for cancer treatment. Their ability to produce ROS is attributed to a small energy gap (ΔE_ST_) between the lowest singlet-excited state (S_1_) and the lowest triplet-excited state (T_1_). The small ΔE_ST_ value is expected to promote the efficient ISC process to enhance ROS generation. Wang et al. (2018) designed compounds, such as TTPy and MeTTPy, with strong electron donor–acceptor (D–A) interactions that could induce the intramolecular charge transfer (ICT) effect, thus leading to smaller ΔE_ST_ (~.277 eV) and bathochromic shifts of absorption and emission. Due to the intrinsic propeller structure of TAA, these compounds turned to be effective PSs with the AIE property and long emission wavelengths. Solutions of TTPy and MeTTPy exhibited a weak fluorescence with emissions at 682 and 687 nm. Due to the presence of a pyridinium salt, such compounds selectively targeted cancer cell mitochondria, as demonstrated by a co-localization experiment upon co-staining with MitoTracker Green. When the AIE molecules entered the tumor sites, they interacted with the tumor cells through electrostatic interactions, and their fluorescence intensity increased accordingly. At low working concentrations, TTPy and MeTTPy exhibited strong fluorescence and excellent photostability, which are the two most important features for imaging guided therapy. After intratumor injection of MeTTPy for 5 min, a strong fluorescent signal was observed at the tumor site, which could last for 24 h.

Most NPs can target tumor sites due to the EPR effect. In addition, NPs can be further functionalized with targeting groups to secure active tumor targeting. Among active tumor targeting strategies, the enzyme-directed self-assembly of AIEgens was conceived based on enzyme overexpression by cancer cells (Ji S. et al., 2018). For instance, folic acid is effective to promote the cellular uptake by folic acid receptor-positive cancer cells, while triphenylphosphine (TPP) drives the accumulation of AIE dots in mitochondria. An AIE PS linked to TPP was reported, which could accumulate in the mitochondria of tumor cells; however, when two triphenylphosphine groups were introduced in the PS, the resulting molecule destroyed the mitochondrial membrane and also acted as a chemotherapeutic drug (Zhang et al., 2015). Both chemotherapeutic drugs and PSs were loaded on NPs and subsequently delivered to tumor cells to induce a synergistic therapeutic effect.

**Figure 4:**
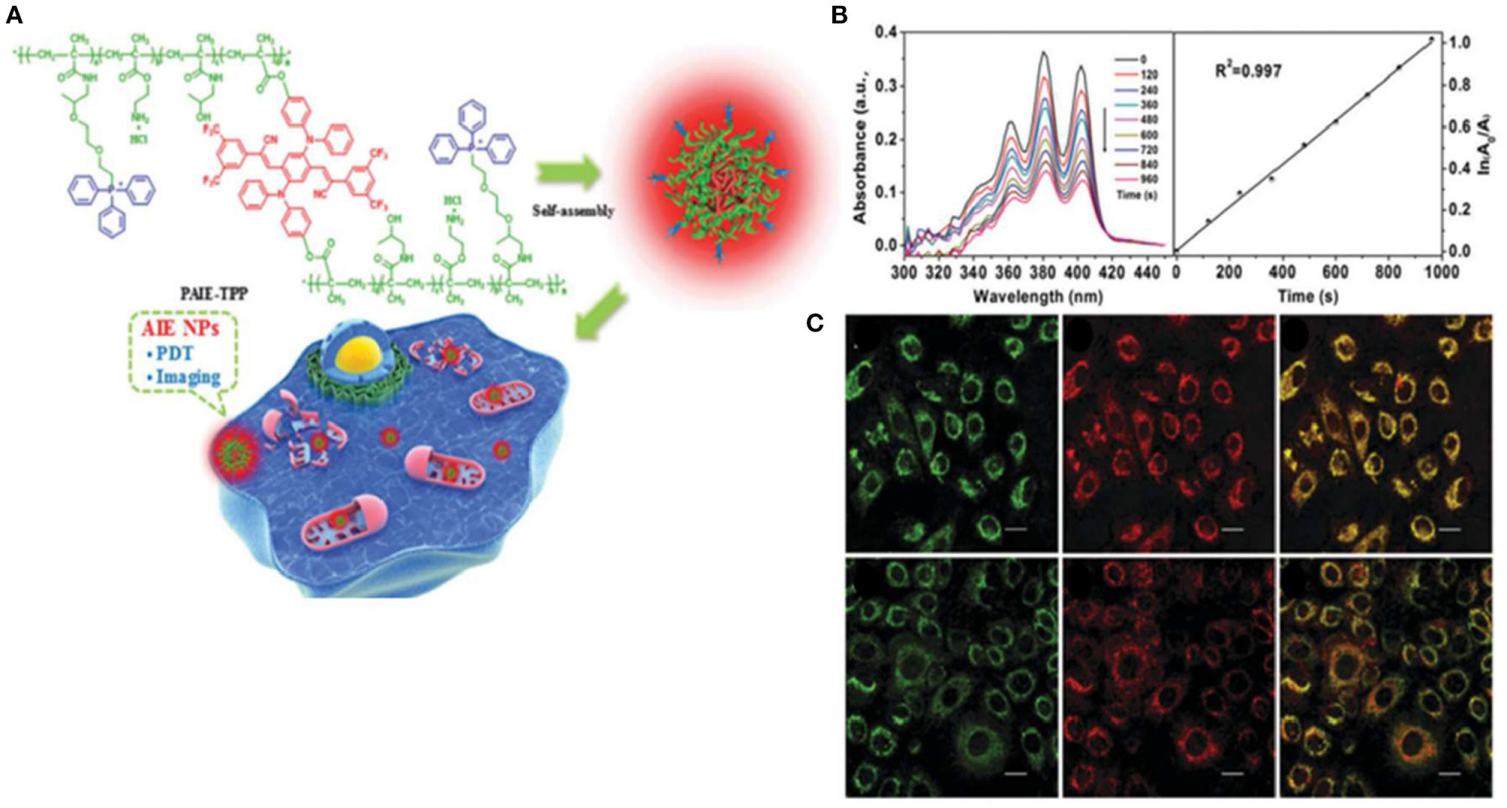
**(A)** Schematic illustration of PAIE-TPP NPs as mitochondria-targeted agents for simultaneous targeted delivery, mitochondrial imaging, and PDT destruction. **(B)** UV–Vis absorbance changes of the ROS indicator ABDA (200 μM) mixed with PAIE-TPP NPs (200 μg/ml) at different light irradiation times and decomposition rates of ABDA. **(C)** CLSM images of A549 cells after incubation with MitoTracker Green (50 nm) and AIE NPs (PAIE-TPP or PAIE, 40 mg/ml). Left: MitoTracker Green channel. Middle: AIE NP channel. Right: Merge channel, λ_ex_ = 488 nm, λ_em_ = 662–737 nm. Mito-Tracker Green, λ_ex_ = 488 nm, λ_em_ = 505–525 nm. Reproduced with permission from Zheng Y. et al. (2017). Copyright 2017 Royal Society of Chemistry.

Zheng Y. et al. (2017) synthesized a NIR-emissive AIEgen, with an emission peak at 647 nm, which consisted of an electron-donating diphenylamine moiety and electron-withdrawing cyano groups. As shown in Figure 4A, self-assembled AIE cross-linked copolymer (PAIE-TPP) NPs are composed of hydrophilic TPP and PEG, a common hydrophilic polymer. The average diameters and surface zeta potentials of PAIE-TPP NPs are 260 nm and +39 mV, respectively. Moreover, the ROS quantum yield was 77.9% under white light irradiation (Figure 4B). As shown in Figure 4C, PAIE-TPP NPs targeted the mitochondria in A549 cells, displaying a Pearson's correlation coefficient of 0.93. A strong ROS generation *in vitro* is observed by increased dichlorodihydrofluorescein (DCF) fluorescence and cytotoxicity study. This review demonstrates PAIE-TPP NPs to be a mitochondrial targeting probe with precise ROS generation at the organelle level.

**Figure 5:**
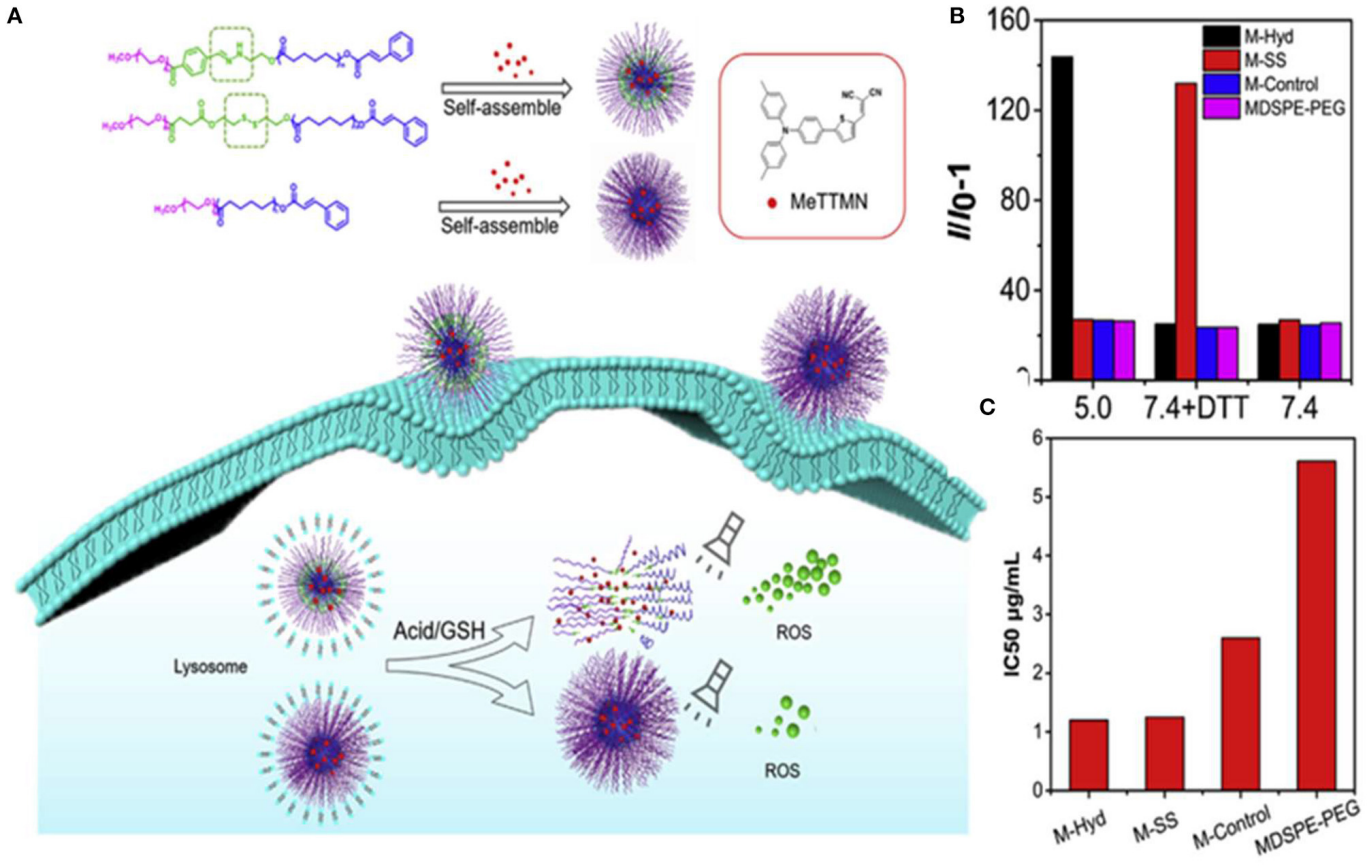
**(A)** Illustration of stimuli-responsive AIE NPs with high ROS generation efficiency and boosted PDT effect. **(B)** Relative change in fluorescent intensity (I/I_0_-1) at 525 nm of dichloro-dihydro-fluorescein (DCFH) with MeTTMN-loaded micelles upon white light irradiation for 10 min. **(C)** The IC_50_ values of MeTTMN-loaded micelles. Reproduced with permission from Li et al. (2020). Copyright 2020 Elsevier.

To improve the PDT efficiency of the drug delivery systems carrying AIE-active photosensitizers, Li et al. (2020) designed two kinds of stimuli-responsive nano-micelles carrying a far-red emissive AIE PS, MeTTMN, namely pH responsive polymers mPEG-Hyd-PCL-CIN (P-Hyd) and redox-responsive polymer mPEG-SS-PCL-CIN (P-SS), which were prepared based on hydrophilic poly(ethylene glycol) (mPEG) and hydrophobic caprolactone (ε-CL), respectively, as shown in Figure 5A. Nano-micelles can successfully carry MeTTMN with high loading efficiency and remarkable stability. The average size of these NPs was around 90 nm with narrow size distribution, smaller than that of commercial MDSPE-PEG. Impressive increase of fluorescence intensity was found upon incubating with pH-responsive M-Hyd at acetate buffered solution (ABS: pH 5) and redox-responsive M-SS at PBS with 10 mM DL-dithiothreitol (DTT), while much less increase of fluorescence intensity was determined in the presence of M-Control and MDSPE-PEG under the same conditions (Figure 5B). The result suggested the excellent performance of stimuli-responsive nano-micelles in terms of ROS-generation efficiency, which was beneficial for PDT applications. Upon white light irradiation for 5 min, a dose-dependent cytotoxicity was found. The IC_50_ values of M-Hyd, M-SS, M-Control, and MDSPE-PEG were determined to be 1.20, 1.25, 2.60, and 5.61 μg/ml, respectively, as shown in Figure 5C. The results of cytotoxicity further confirmed that AIE PSs released from nanoparticles could enhance ROS generation efficiency and the PDT effect. Hence, the stimuli-responsive nano-micelles have great potential as carriers to deliver AIE PSs for boosted PDT effect.

## Others

In addition to TPE and TAA, AIE PSs, which compromise other chemical structures, can also perform well in cancer theranostics. Chen and Chang (2016) synthesized a molecule named NV-12P, which combined a 4-vinylpyridinium moiety with a cation aliphatic side chain. After light exposure, the fluorescence intensity of dichloro-dihydro-fluorescein diacetate (DCFH-DA) at 525 nm was over 100-fold higher than that before irradiation. In addition, the green emission from DCF was observed to increase after light irradiation for 1 min. These results proved that NV-12P was able to generate ROS upon light irradiation both in the solution and in cells. NV-12P exhibited an excellent selectivity for cancer cells over normal cells. After being irradiated with either a visible light source (400–700 nm) or an additional UV-A light source (average of 20 mW/cm^2^, 340–700 nm), the EC50 values were ~6.5 and ~4.5 μM for in HeLa and CL1-0 cells, respectively, while no obvious photo damage to MRC-5 normal cells MRC-5 was observed.

## Combined Therapies Involving AIE Dots

In order to improve its anticancer efficacy, many researchers have combined PDT with other therapies such as chemotherapy (Yuan et al., 2015; Zhang et al., 2015), photothermal therapy (PTT) (Feng et al., 2016; Guo et al., 2016), gene therapy (Jin et al., 2016), and immunotherapy (Lu et al., 2016; Xu et al., 2017), in order to achieve a synergistic effect for cancer treatment. AIE dots showed excellent performance in combination therapies, usually with a synergistic effect of “1 + 1 > 2.”

## Combined Therapy of PDT and Chemotherapy

Chemotherapy represents one of the first-line clinical treatment methods for cancer (Pérez-Herrero and Fernández-Medarde, 2015; Wagner et al., 2017); however, long-term chemotherapy is prone to multidrug resistance, so that the use of a single chemotherapeutic drug is not typically favored (Wang et al., 2015; Cui et al., 2018). The emergence of non-cross-resistant chemotherapeutic drugs could provide an effective solution to this drawback (Nagy et al., 2019). For example, cisplatin prodrugs were developed by linking an AIE PS (TPE derivative) and doxorubicin (DOX) (Yuan et al., 2014). After entering the cancer cells, DOX could be released and the AIE molecules were separated from it, hence restoring the fluorescence. In this way, AIE molecules were used for both diagnostic and therapeutic purposes, yielding a good synergistic effect together with that of the two chemotherapeutic drugs (Liang et al., 2015; Guo et al., 2020). In another case, a cisplatin prodrug and AIE PS were co-loaded into nanocarriers and delivered to tumor sites by the EPR effect; later, both the drug and the AIE PS were released and functioned synergistically (Wang et al., 2015; Yuan et al., 2015).

A series of compounds containing artemisinin (ART), an antiapoptotic inhibitor, or TPETH, and AIE PS were synthesized by Feng et al. (2018). The effect of both TPETH-Mito-1ART and TPETH-Mito-2ART was significantly improved with synergistic effect. TPETH-Mito-1ART showed efficient intracellular ROS generation, as well as mitochondrial targeting. Furthermore, the delivery of ART to mitochondria also quickly promoted its anticancer activities. Jiang et al. (2016) designed a quaternary ammonium-modified tetraphenylethylene derivative (QA-TPE) as a chemotherapeutic agent, which could also guide the therapy through targeted cell imaging by its turned-on fluorescence. Hyaluronic acid (HA) was used as the aggregation-inducing scaffold as well as the tumor-targeting agent. The endogenous Haase in CD44 receptor-mediated cancer cells triggered the release of QA-TPE for imaging-guided hysteretic chemotherapy activity.

**Figure 6:**
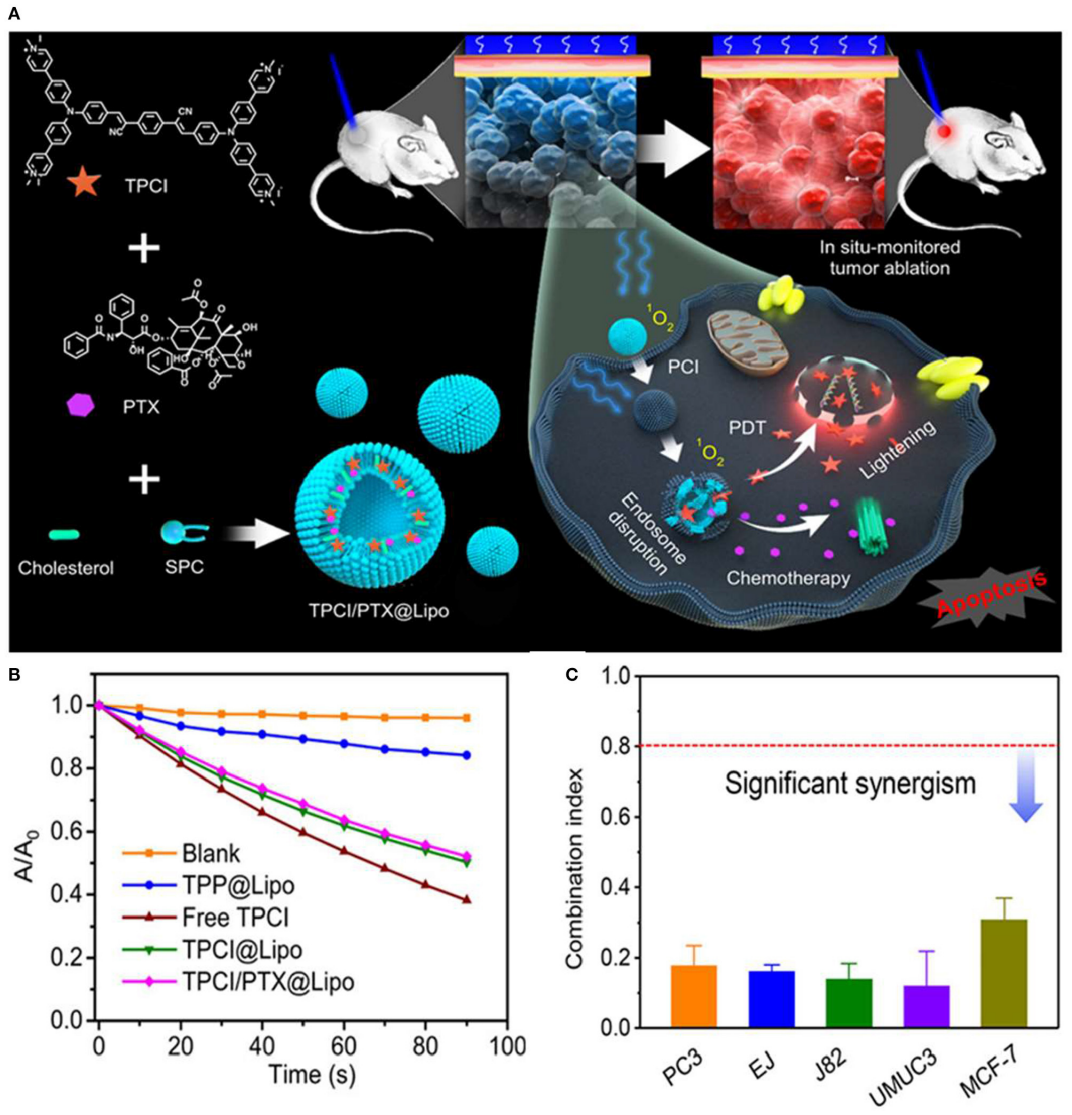
**(A)** Schematic illustration of paclitaxel (PTX)-potentiated TPCI-based photodynamic theranostics for synergistic ablation of large tumors and self-reporting of the anticancer effect. **(B)** Absorption changes of ABDA in the presence of free TPCI and various liposomes under blue light irradiation in water. **(C)** The combination index (CI) values of TPCI/PTX@Lipo in treating various cancer cells (460 nm, 1 mW cm^−2^, 10 min). Reproduced with permission from Wang et al. (2020). Copyright 2020 American Chemical Society.

Wang et al. (2020) developed a liposome-based AIE dot, containing both TPCI, an AIE PS with superior ROS generation efficacy, and a chemotherapy agent, paclitaxel (PTX) (Gao et al., 2019). The AIE dot TPCI/PTX@Lipo, with an average size of 100–120 nm, exhibited a superb synergistic anticancer effect against tumor cells (Figures 6A,B). Moreover, the cytotoxicity of TPCI/PTX@Lipo was significantly higher than that of liposomes loaded with a single drug, where the IC_50_ values of both drugs had been reduced in a range of 5- to 30-fold in treating carcinoma cell lines compared to single chemotherapy (PTX@Lipo) or PDT (TPCI@Lipo), even at low irradiation fluence. The combination index (CI) values of TPCI/PTX@Lipo were below 0.5 for examined cell lines, as shown in Figure 6C. The effective ablation of large tumors evidenced the pronounced potentiation effect of PTX on PDT and the synergistic antitumor effect of the combined therapy *in vivo*. Surprisingly, the released TPCI could instantly fluoresce in the nuclei of dead cells and self-report the precise therapeutic effect in real time. More strikingly, TPCI/PTX@Lipo could report the therapeutic effect by lighting up the nuclei of dead cells triggered by both PDT and the combined therapeutic method.

## Combined Therapy of PDT and PTT

As a light-controllable and non-invasive therapy, PTT has aroused increasing attention for tumor ablation. To avoid the hypoxic microenvironment for PDT and acquired heat shock effect in PTT, the cooperation of PDT and PTT is considered as a synergistic effect with improved therapeutic outcomes. Many researchers encapsulate photothermal reagents and photosensitizers into nanoparticles to achieve synergistic treatment of tumor cells. A remarkable feat is that some AIE dots can not only produce ^1^O_2_ but also have heat generation efficiency.

**Figure 7:**
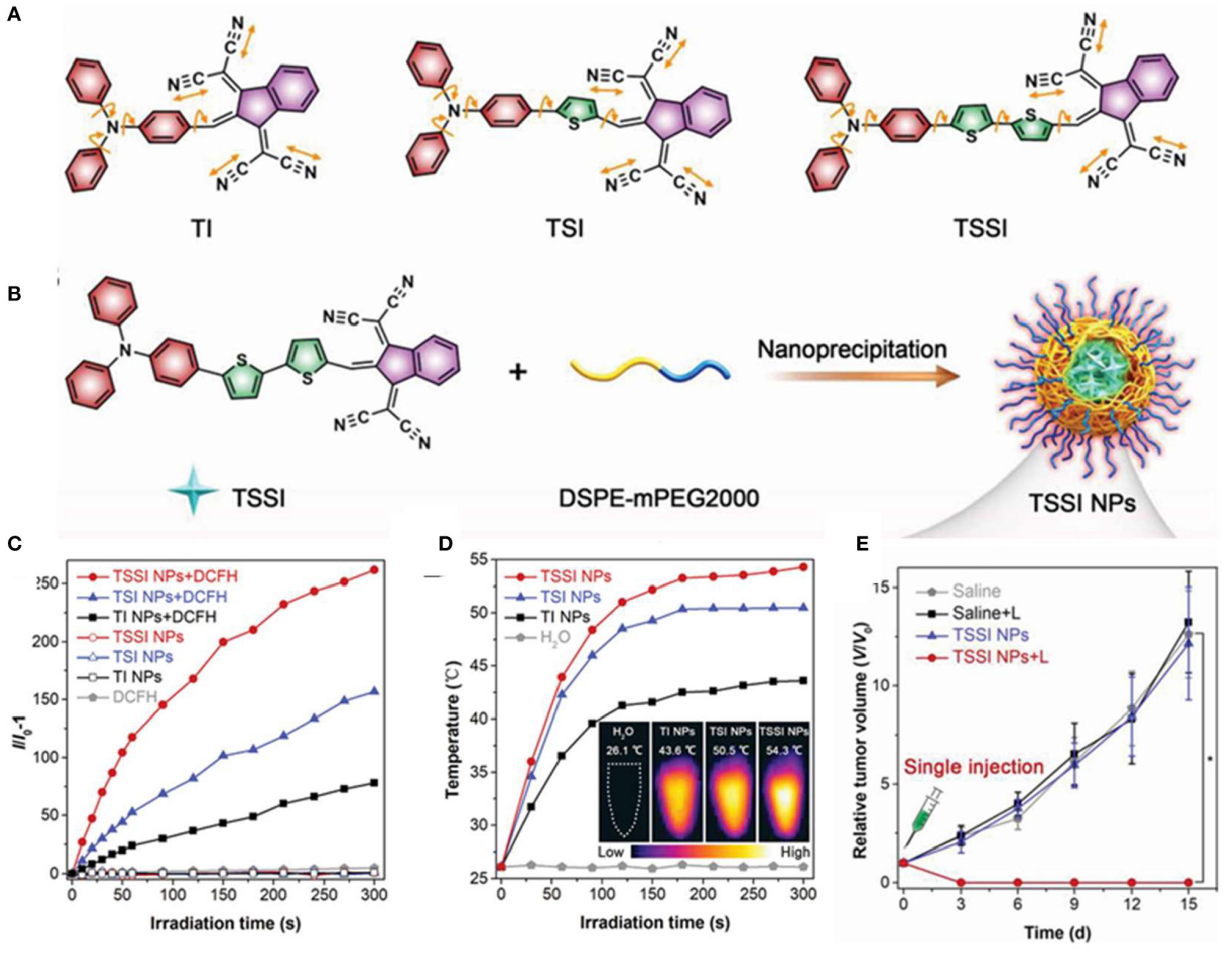
**(A)** Molecular structures of designed compounds including TI, TSI, and TSSI. **(B)** Preparation of TSSI NPs by a nanoprecipitation method. ROS generation **(C)** and the photothermal conversion behaviors **(D)** of these NPs in aqueous solution under 660 nm laser irradiation. Insert: The corresponding IR thermal images of these NPs samples at 5 min post-irradiation. **(E)** Time-dependent tumor growth curves of tumor-bearing mice with various treatments (*n* = 6, *p < 0.001). Reproduced with permission from Zhang et al. (2020). Copyright 2020 Wiley Online Library.

Zhang et al. (2020) reported that AIE dots displayed NIR-II fluorescence signals centered at 992 nm and high ROS and heat generation efficiency. They designed three AIEgens with different thiophene segments in the conjugated backbones, named TI, TSI, and TSSI. To fabricate biocompatible and well-dispersible AIE NPs, hydrophobic TSSI were directly encapsulated with amphiphilic co-polymer DSPE-mPEG2000, as shown in Figures 7A,B. TSSI NPs had average sizes of about 55.5 nm and uniform spherical morphology measured by DLS and TEM. The emission intensity of DCFH reached over 250-fold after 5 min of laser irradiation, and the ROS production efficiency of TSSI NPs was much superior to those of TI and TSI NPs, attributing to the stronger D–A interaction of TSSI (Figure 7C). Moreover, the photothermal effect of TSSI NPs was obvious with plateau temperatures of 54.3°C after 5-min irradiation of NIR laser (Figure 7D). At 12 h post-injection, intensive NIR-II fluorescence and photoacoustic (PA) signals were observed in the tumor region with affordable simultaneous accurate tumor imaging and complete tumor elimination. Due to the excellent synergistic efficiency of PDT and PTT, the tumors were completely extinct without any recurrence, leaving only a scar at day 15 after treatment with only a single injection and NIR irradiation for one time at 12 h post-injection, as shown in Figure 7E.

An AIEgen, named TFM, with photothermal conversion efficiency of 51.2% was reported recently (Wang et al., 2019). Moreover, TFM NPs showed excellent PA effect and effective ROS generation. TFM NPs have uniform spherical morphology with a mean diameter size of 72 nm measured by TEM and show excellent stability at physiological condition. The temperature of TFM NPs dispersion was rapidly raised by using 3 mg/ml of TFM NPs, increasing from 24.4 to the plateau at 65.3°C in 10 min. Meanwhile, an obvious PA signal of TFM NPs dispersion with a good linear relationship was detected. The evaluation of ROS generation efficiency of TFM NPs was conducted by using DCFH-DA as an indicator, and the result showed 16-fold enhancement of emission intensity of DCFH-DA. *In vivo* evaluation showed that the tumor can be detected by the PA signal of highly accumulated TFM NPs, and the tumor growth of phototherapy groups involving both TFM NPs and laser irradiation were suppressed significantly.

## Combined Therapy of PDT and Radiotherapy

Radiotherapy is another important clinical treatment method for tumors (Atun et al., 2015). It can inhibit tumors locally and has a good therapeutic effect in combination with surgical treatment (Stokes et al., 2018). However, due to the resistance of certain tumor cells to radiation, the therapeutic effect may be greatly restricted (Kelland, 2007). Researchers used radiosensitizers, such as small molecules and Au NPs, to make tumor cells more sensitive to radiation for improved RT (Adams et al., 2016). Some studies have shown that mitochondria are highly correlated with radiation resistance (Wu et al., 2016).

In order to improve RT efficacy, Yu et al. (2017) first proposed that AIE-gens could target tumor sites and sensitize the corresponding tumor to the ionizing radiation under white light illumination. After A549 cells were incubated with diphenylamino (DPA-SCP) for 2 h and illuminated with white light for 1 min, a significant amount of ROS was generated. PDT by using DPA-SCP alone was not able to kill cancer cells efficiently at low concentrations or irradiation doses. Surprisingly, DPA-SCP significantly killed tumor cells in combination with ionizing radiation, as shown by a clonal survival assay. DPA-SCP could make A549 cancer cells sensitive to the ionizing radiation and had a synergistic effect of “1 + 1 > 2” for cancer treatment.

Radiodynamic therapy (RDT), which can overcome the drawbacks of the limited penetration depth for PDT and the scattered energy in the tumor for RT, is a kind of method based on x-ray-induced dynamic therapy (Sun et al., 2020). Hf-AIE coordination polymer nanoparticles (CPNs), based on Hf^4+^ and an AIE PS of TPEDC-DAC, showed both strong RT and RDT effects under x-ray irradiation (Liu et al., 2021). TPEDC-DAC NPs with the generated ROS upon light or x-ray irradiation overcome the inherent drawback of short excitation wavelength at 380 nm.

Besides, Chen et al. (2019) reported that mitochondrial targeted AIE photosensitizers generated focused oxidative stress under light excitation, which is very efficient to induce greatly amplified immunogenic celldeath.

Currently, PDT is limited to subcutaneous tumors due to the insufficient penetration depth of excitation light in most cases. Clinically, PDT is mostly used in the treatment of skin diseases and superficial tumors thanks to the advantages of high safety, less trauma, and fewer adverse reactions. Besides, some AIE PSs can be used for fluorescence imaging guidance in surgery.

## Conclusion and Perspectives

The discovery of the AIE phenomenon has stimulated the development of novel PSs with enhanced performance for their applications, especially in view of their role in PDT. Specifically, AIE dots show improved efficiencies in the aggregated state as well as within nanocarriers. Furthermore, AIE dots exhibit better photostability and ROS generation ability; thus, effectively overcoming the limits of traditional PSs and opening a new avenue for PDT applications. Since AIE PSs can be functionalized with different functional moieties, many AIE PSs bearing various targeting or other responsive groups have been designed and synthesized. The AIE property endows the corresponding PSs with the ability to efficiently function in a complicated physiological environment.

Although AIE dots have greatly inspired the application of AIE PSs in PDT, some important challenges in this field still remain unsolved. First, the ability of tumor targeting remains a challenge. AIE dots are generally efficient in ROS generation; however, the specificity remains a major issue especially when dots are administrated by intravenous injection precisely. Secondly, to achieve PDT with AIE PSs *in vivo*, the design of novel AIE PSs with ultra-bright NIR emission, especially in the NIR-II region, is urgently needed; however, this remains a great challenge since long excitation wavelengths usually result in decreased FL intensity and ROS quantum yield. This conflict is still hindering the development of ideal AIE dots. It is necessary to develop some AIE PSs that compromise both the ROS quantum yield and NIR properties. In addition, although many AIE PSs have been developed in recent years, they are still based on classical AIE core structures such as TPE and TPA. AIEgens with other structures, such as siloles, phosphole oxides, and their derivatives, have not been used in PDT (Chen et al., 2014, 2016; Zhuang et al., 2017). Therefore, there are more opportunities to modify these molecules with D–A–D structures to approach a small ΔE_ST_ value or to conjugate them with a photosensitizer of high quantum yields of singlet oxygen generation. Furthermore, the biodegradable and the long-term side effects of AIE dots remain unclear. Finally, regulating the tumor microenvironment may have greater research prospects than directly killing cancer cells. Despite the PDT elimination of tumor cells, ROS-related tumor microenvironment could be a target for AIE dots. Due to the hydrophobic nature of most AIEgens, supramolecular assembly is a meaningful tool to construct AIE dots governed by non-covalent interactions with AIEgens. AIE dots with different chemical structures and properties should be considered separately. In terms of loading efficiency, it is good to choose nanocarriers that interact with AIE molecules through hydrophobic and electrostatic interactions, hydrogen bonding, and cation–π interaction. In terms treatment effects, nanocarriers with different particle sizes, tumor-targeting properties, and stimuli-responses are good choices. AIE dots with selective stimuli responsiveness to tumor related microenvironment are still in the early stage. Regulating the ROS of tumor immune microenvironment produced by AIE dots maybe an excellent choice.

Overall, multifunctional PSs with AIE characteristics are shedding new light on anticancer PDT. Many AIE dots are being exploited for better performance in the PDT treatment of tumors. As the AIE field undergoes rapid development and the limiting factors are gradually addressed, the potential to utilize AIE dots for clinical PDT is boosting.

## Author Contributions

ZH and ST wrote the manuscript. FM and LL edited the manuscript and provided theoretical guidance. All authors contributed to the discussions of the content of this review.

## Conflict of Interest

The authors declare that the research was conducted in the absence of any commercial or financial relationships that could be construed as a potential conflict of interest.
